# Solar superstorm of AD 774 recorded subannually by Arctic tree rings

**DOI:** 10.1038/s41467-018-05883-1

**Published:** 2018-08-28

**Authors:** J. Uusitalo, L. Arppe, T. Hackman, S. Helama, G. Kovaltsov, K. Mielikäinen, H. Mäkinen, P. Nöjd, V. Palonen, I. Usoskin, M. Oinonen

**Affiliations:** 1 Finnish Museum of Natural History, University of Helsinki, P.O. Box 64, 00014 Helsinki, Finland; 2 Department of Physics, University of Helsinki, P.O. Box 64, 00014 Helsinki, Finland; 3 Natural Resources Institute Finland, Eteläranta 55, 96300 Rovaniemi, Finland; 4 Ioffe Physical-Technical Institute, Politekhnicheskaya 26, 194021 St. Petersburg, Russia; 5 Natural Resources Institute Finland, Tietotie 2, 02150 Espoo, Finland; 6 Space Climate Research Unit and Sodankylä Geophysical Observatory, University of Oulu, Pentti Kaiteran katu 1, 90014 Oulu, Finland

## Abstract

Recently, a rapid increase in radiocarbon (^14^C) was observed in Japanese tree rings at AD 774/775. Various explanations for the anomaly have been offered, such as a supernova, a γ-ray burst, a cometary impact, or an exceptionally large Solar Particle Event (SPE). However, evidence of the origin and exact timing of the event remains incomplete. In particular, a key issue of latitudinal dependence of the ^14^C intensity has not been addressed yet. Here, we show that the event was most likely caused by the Sun and occurred during the spring of AD 774. Particularly, the event intensities from various locations show a strong correlation with the latitude, demonstrating a particle-induced ^14^C poleward increase, in accord with the solar origin of the event. Furthermore, both annual ^14^C data and carbon cycle modelling, and separate earlywood and latewood ^14^C measurements, confine the photosynthetic carbon fixation to around the midsummer.

## Abstract (web summary)

Tree rings retain information of sudden variations of ancient radiocarbon (^14^C) content, however the origin and exact timing of these events often remain uncertain. Here, the authors analyze a set of Arctic tree rings and link a rapid increase in ^14^C to a solar event that occurred during the spring of AD 774.

## Introduction

Radiocarbon (^14^C) is produced in the Earth’s stratosphere/troposphere mostly by thermal neutrons captured by nitrogen nuclei. Thermal neutrons are produced by nuclear reactions of high-energy cosmic rays, originating mainly from galactic sources, or to a smaller extent, from the Sun. ^14^C oxidizes to CO_2_, and tropospheric CO_2_ is eventually stored in tree rings through CO_2_ fixation by photosynthesis during growing season. Rapid events producing ^14^C are eventually reflected in tropospheric or tree-ring ^14^C measurements as peaks with an abrupt increase and a long decrease of dozens of years caused by the exchange of ^14^C between the atmosphere and Earth’s carbon reservoirs. Such a ^14^C increase was recently observed in Japanese tree rings by Miyake et al.^1^ from AD 774 to AD 775 (henceforth ME meaning Miyake event), followed by similar observations throughout Northern Hemisphere (NH)^2–6^ and even in New Zealand^7^.

The shielding effect of the geomagnetic field against charged cosmic ray particles, i.e., the geomagnetic (vertical) cutoff rigidity (*R*_C_) is the highest near the equator and decreases towards the polar regions. Therefore, particles producing cosmogenic nuclei most easily penetrate into the atmosphere at high geomagnetic latitudes^8^. In addition to stratospheric production, a fraction of ^14^C can be formed in the troposphere allowing for rapid photosynthetic fixation.

The initial atmospheric ^14^C pool is affected by carbon cycle dynamics. In addition to vertical transport, horizontal transport of ^14^C within the atmosphere alters the local ^14^C pool sampled by the trees. However, remnants of the original polar production and its latitudinal dependence may still be observed in the ^14^C signal stored into tree rings by the photosynthetic assimilation, particularly due to tropospheric production of ^14^C. Indeed, such subtle latitudinal differences induced by the initial production and entangled with the carbon cycle dynamics have been observed by measuring the tree-ring ^14^C contents^9,10^. Assuming a solar origin for ME, and thus initial ^14^C production at polar latitudes, one may observe changes in the intensity of the anomaly according to the latitude.

Here, we report the measurement of annual ^14^C content of a Finnish Lapland tree during the ME, so far closest to the contemporary Geomagnetic North Pole (GNP). By utilizing a peak-fitting approach and complementing our results with previously published data, we find a latitudinal tendency in the event intensities, which is consistent with a solar origin. Furthermore, based on both carbon cycle modelling and additional earlywood (EW) and latewood (LW) ^14^C tree-ring measurements, we find that the event occurred probably during the spring of AD 774.

## Results

### ^14^C intensities

**Fig. 1:**
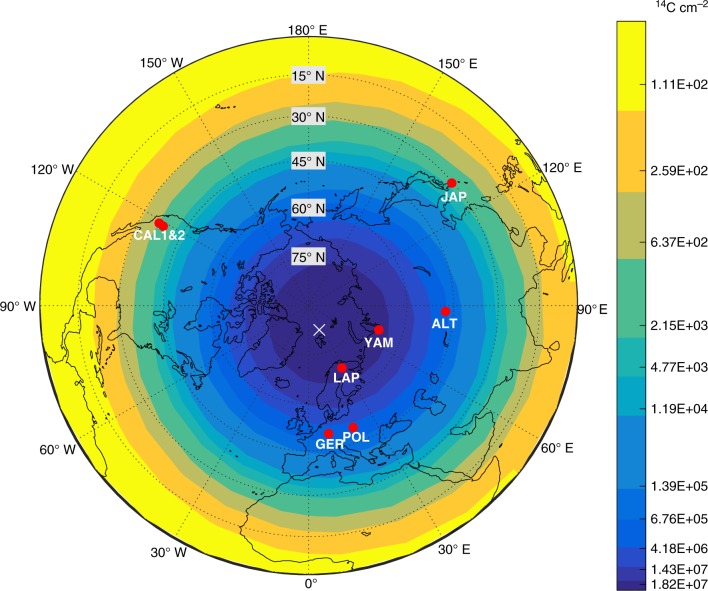
Map of the study region. The colour codes show the latitudinal differences of ^14^C production rate (^14^C cm^−2^) for a typical strong SPE (such as 23 February 1956, the strongest directly observed event, Supplementary Table 2). The ^14^C production model^36^ is described in the Methods section. The red full circles with three-letter coding show the measurement locations for ^14^C intensities (see text). The cross illustrates the location of the Geomagnetic North Pole in AD 775

For a strong Solar Particle Event (SPE), like that of 23 February 1956, the atmospheric production of ^14^C can be orders of magnitude larger in polar regions compared to tropical latitudes, with high *R*_C_’s (Fig. 1). Hence, we hypothesize that the ^14^C intensity induced by an ME has a latitudinal tendency if the event is of solar origin because *R*_C_ governs the number of solar particles that enter the Earth’s atmosphere and the tropospheric production supports rapid photosynthetic fixation. The *R*_C_ values, calculated using the recent archaeomagnetic model AF_M^11^ (see Methods), for the epoch of AD 775 provide insight into the initial ^14^C production rates according to locations (see Supplementary Table 1). The location of the GNP at AD 775 (GNP_AD775_) can be estimated to be north-east from Svalbard at approximately 82° N, 25° E. In the vicinity of the GNP_AD775_, the modelled *R*_C_ approaches 0 GV, implying that even low-energy particles can impinge on the atmosphere. Suggested hard particle spectrum of ME^12^ would likely result in partial tropospheric production of ^14^C (see Methods).

The hypothesis is tested by evaluating the significance of the latitudinal trend of the available ME data through Pearson’s correlation. Our tree-ring material is derived from a precisely cross-dated 7600-year long Scots pine (*Pinus sylvestris*) record collected from Finnish Lapland^13–15^, in particular, from Lake Kompsiojärvi (68.5N, 28.1E; hereafter LAP). We complement our results with previously published and precisely cross-dated data from Yamal/Russia (67.5° N, 70.7° E; YAM)^3^, Poland (50.1° N, 20.1° E; POL)^4^, Germany (50° N, 9° E; GER)^2^, Altai/Russia (47.5° N, 87.5° E; ALT)^5^, Japan (30.4° N, 130° E; JAP)^1^, USA (38.3° N, 118.7° W; CAL1^3^ and 36.5° N, 118.8° W; CAL2^6^) and New Zealand (36.0° S, 173.8° E; NZL)^7^ to analyze the latitudinal behaviour. The LAP and YAM locations are within 15° of latitude from the GNP_AD775_. This close vicinity is reflected as low modelled vertical cutoff rigidities of *R*_C, LAP, YAM_ ~ 0.1 GV. At the southern extreme of the locations (CAL1, CAL2, JAP), the values are very high (*R*_C, CAL, JAP_ ~10 GV), which should yield drastically lower initial atmospheric production of ^14^C, particularly in these locations (Fig. 1). We utilize peak-fitting analysis to extract the full ^14^C intensities related to ME to acknowledge the temporal spread of tropospheric ^14^C increase into several years (see Methods).

**Fig. 2:**
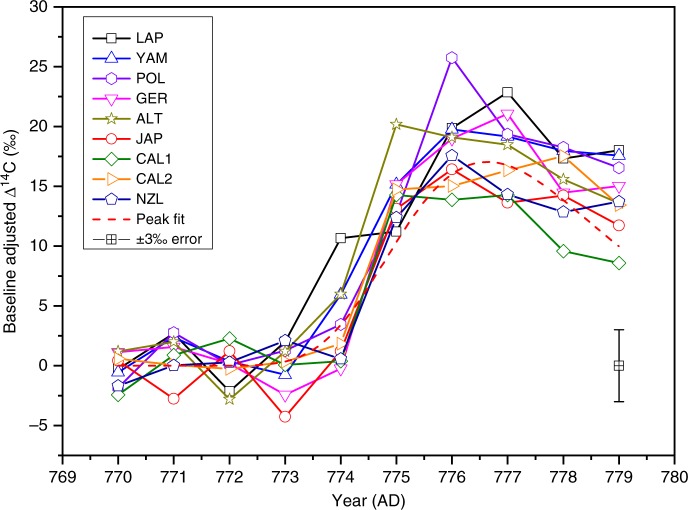
^14^C measurements of the ME from multiple locations (see main text above for abbreviations). The horizontal axis represents the calendar year of the growing season, and the vertical axis represents the age-corrected and baseline-adjusted Δ^14^C (see Methods, Supplementary Table 3). The baseline adjustment has been performed for easier comparison of the data. The dashed red line shows an example of the analytical type I Gumbel distribution function (GDF) fit to the JAP data. Each measurement has a standard error of typically ±3‰ as visualized in the lower right corner. Original intensities, uncertainties and fitting procedure are as described in the Methods section

**Fig. 3:**
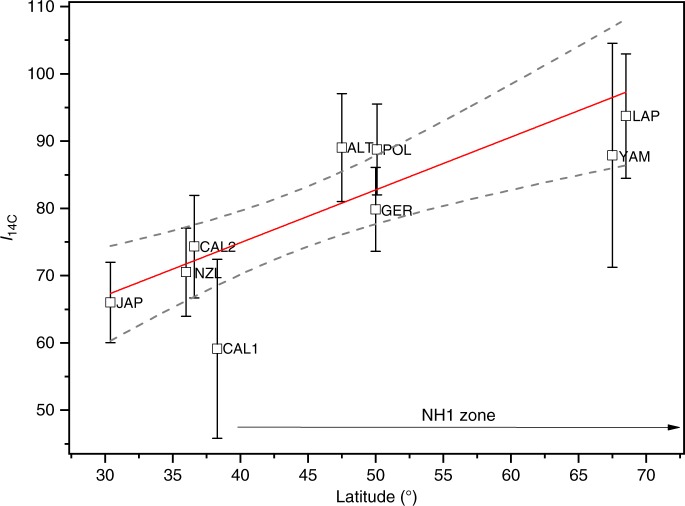
Latitude vs. the ^14^C intensity *I*_14C_. The red line is a linear fit to the data. The uncertainties are based on error propagation and represent one standard error, and the dashed lines indicate the 95% confident intervals for the fit. The ^14^C intensities are obtained by fitting a GDF with each data set and by defining the *I*_14C_ as an integral of this curve (see Methods). NH1 zone has been adopted from Hua et al.^18^. Differences in the ^14^C spatial distribution are manifested by distinct zones that span through both hemispheres, latitudes North of 40°N (NH1 zone) having the highest bomb-peak ^14^C intensities

We observe a clear increase of ^14^C content in subfossil tree rings from LAP (Fig. 2, Supplementary Table 3) corresponding to ME. Actually, the ^14^C content is increased already during AD 774—it differs 5.3*σ* from the averaged ^14^C background of AD 770–773. Interestingly, YAM also shows an increase during AD 774 with respect to its ^14^C background. This increase is not statistically different from LAP (*p* = 0.24, Student’s *t*-test) reflecting their small latitudinal separation. The deduced ^14^C intensities *I*_14C_ (see Methods, Supplementary Table 1) as a function of latitude are shown in Fig. 3. Our null hypothesis of a constant *I*_14C_ (slope = 0) as a function of latitude is rejected with a significance level of *p* = 0.002 (Student’s *t*-test). Thus, inarguably, there is a statistically significant latitudinal trend within the data. The relation between the latitude and ^14^C intensity is supported by the high correlation of *r*(lat, *I*_14C_) = 0.87. This latitudinal trend cannot be explained by a possible seasonal variation of the tropospheric ^14^C concentration nor by differing growing season lengths. Although some seasonal variation may occur after an instant injection of ^14^C into the atmosphere^16^, we found its role insignificant (see Supplementary Note 1). The observed trend is consistent with the higher production rate of ^14^C at high latitudes and its redistribution within the atmosphere towards lower latitudes and into other carbon reservoirs. This finding implies a polar enhancement of the cosmogenic signal, which is also experimentally known for ^10^Be in polar ice^17^. The enhancement is probably mediated partially by the direct tropospheric production of ^14^C. The polar enhancement shown here supports our hypothesis of a charged-particle-induced ^14^C increase.

Interestingly, differences within the NH Zone 1 (NH1, Fig. 3) locations (LAP, YAM, POL, GER, ALT) are relatively small, although visible. However, there are larger differences between the intensities of NH1 and non-NH1 locations (JAP, CAL1, CAL2, NZL). In fact, we see statistically significant (*p* = 0.008, permutation test) difference between NH1 and non-NH1 zones (see Methods). This indicates similar atmospheric circulation patterns defining the ^14^C spatial distributions for ME as for the bomb peak induced by atmospheric nuclear detonations in NH, and probably reflects the locations in both sides of the tropospheric Ferrel cell–Hadley cell boundary^18^.

### Origin of the event

The charged-particle flux cannot be due to Galactic Cosmic Rays (GCR) because they would not result in such a peaked ^14^C distribution^19^. This is due to perturbation of GCR by galactic magnetic fields during their travel to Earth causing dispersion and retardation. Moreover, GCR vary within the 11-year solar cycle due to the heliospheric modulation, which is too slow to produce a sharp peak. The observation also excludes the possibility of the increase being caused by supernova γ-rays^1^ or γ-ray bursts^20,21^ because they are not affected by the geomagnetic field and hence would not cause latitude-dependent effects. Furthermore, significant ^10^Be signal linked to the ME was found in both Greenland and Antarctic ice cores^12,22^. In addition, the observed *I*_14C_ of NZL (Fig. 3) is consistent with bipolarity. Such bipolar effects should not occur if the isotope production was confined to only one of the hemispheres. Recently, a cometary impact was suggested to explain the anomaly^23^. However, a comet could explain the observed latitude effects only if it disintegrated near the GNP_AD775_. Even then, it should not cause any signal in ^10^Be. Moreover, due to the required massive size, the comet would have caused dramatic consequences to the biosphere and would not have gone unnoticed^24^.

Recent observations by the Kepler space telescope have shed light on the occurrence rate of these superflares on solar-like stars^25,26^. Maehara et al.^27^ analyzed the Kepler data with a high time resolution to also include shorter duration superflares. Their analysis suggests that the occurrence rate of solar flares with energies ranging from 10^33^ erg to 10^34^ erg are consistent with the ME. On the other hand, it is still unclear whether superflaring stars can be directly compared with the Sun^28^. The Sun’s relatively low magnetic activity around the time of the ME could be seen as problematic for the solar explanation^29^. However, Kilpua et al. recently found that the most extreme geomagnetic storms do not correlate well with the overall solar activity^30^. Hence, based on our observations and the above-described arguments, we suggest the Sun to be the cause of the event, in agreement with the recent study of Mekhaldi et al.^12^ using multiple cosmogenic nuclide records.

### Timing of the event

Dynamics of ^14^C can be elucidated through atmospheric Brewer–Dobson circulation model. Stratospherically produced ^14^C intrudes into troposphere preferentially at mid-latitudes within residence times of 1–2 years whereas mixing of tropospherically produced ^14^C occurs within months^31^. Therefore, the latter may contribute to the observed signal immediately, whereas the assumed mid-latitude intrusion of air from stratosphere to troposphere takes place over in a longer time-scale in shaping up the observed ^14^C peak shape. Our adopted 11-box carbon cycle model (CCM, see Methods), accompanied with data on both growing season length and monthly thermal-time sums (TTS) (see Methods) to mimic the tree-ring ^14^C contents, allows us to reproduce the experimentally observed ^14^C peak shape generally with high correlation. Therefore, we argue that our CCM, although not containing the horizontal carbon cycle dynamics, provides us a reasonable tool to evaluate the event timing, motivated by the observation of ^14^C increase in LAP already at AD 774.

The LAP site is closest to the polar origin of the charged-particle induced ^14^C production. Furthermore, for *P. sylvestris*, carbon isotope signals of fresh photosynthates and tree-ring cellulose correlate strongly, indicating rapid transfer of atmospheric carbon into tree stem^32^. Therefore, Arctic trees of LAP are presumably able to rapidly record the assumed partial tropospheric production. In addition, the short growing season should sensitively probe the ME timing. We define the ME timing as the moment when the tropospherically produced ^14^C has oxidized to ^14^CO_2_ being thus available for photosynthesis (see Methods). We modelled the tropospheric ^14^C increases using different ME timings and compared those to the yearly data of LAP to obtain the best match. Based on Mekhaldi et al.^12^ and in line with Usoskin et al.^2^, we assumed a hard spectrum for the SPE with 70% of the ^14^C produced in the stratosphere and 30% in the troposphere (see Methods). The lowest residual sum of squares (RSS) between the measured and modelled data was observed assuming the ME timing during June/AD 774 (see Methods). Hence, a midsummer timing is implicated. Differing assumed tropopause height profiles can probably result in systematic shifts up to few months, particularly towards spring (see Supplementary Note 2).

**Fig. 4:**
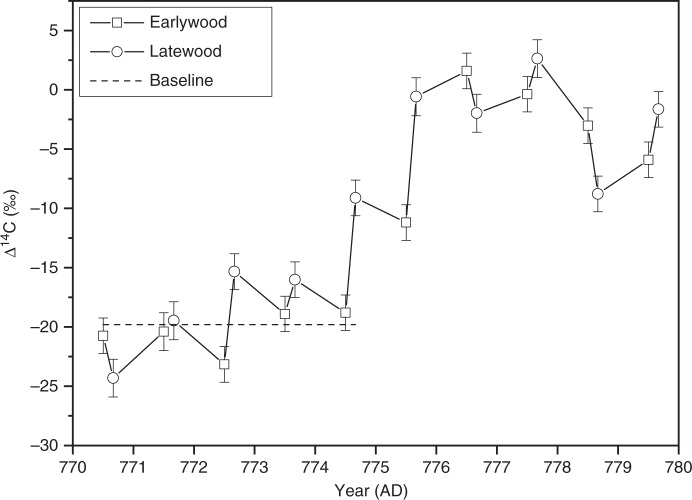
Early- and latewood ^14^C measurements of the ME. The horizontal axis represents the calendar year of the growing season, and the vertical axis represents the age-corrected Δ^14^C. To visualize the average temporal difference between early- and latewood growth, the data points are set to June and August, respectively, for each calendar year. The baseline is defined as the average ^14^C of AD 770–773. The uncertainties are based on ^14^C counting statistic and error propagation and they represent one standard error

The thermal growing season in LAP extends from May to September (see Methods). The EW to LW transition for *P. sylvestris* at these latitudes occurs typically around mid-July^33^, which is close to the ME timing indicated above. Therefore, assuming the ME timing around midsummer in AD 774, only the LW ^14^C content should be elevated in the cellulose formed in AD 774. We tested this hypothesis by EW–LW measurements (Fig. 4) to confirm the ME timing. As expected, both EW and LW data show the similar peak shape as the annual LAP data (Fig. 2), consistent also in magnitude. However, only LW shows a significant (7.1*σ*) increase of the ^14^C content in AD 774, in accord with our expectations. During AD 772, the EW ring width is exceptionally wide (Supplementary Table 3). Therefore, the annual ^14^C signal resembles that of EW_AD772_. Based on large EW/LW ring width ratio, similar mechanism may also be seen in AD 775. Differences between annual and EW/LW signals will be addressed in forthcoming papers based on stable isotopic and ^14^C analyses.

Based on above, the observed ^14^C increase in LW_AD774_ is consistent with our annual data and confirms our model-based result of the event timing. Altogether, observing elevated ^14^C content in LW_AD774_ and not observing it in EW_AD774_ confines the event timing probably to the advent of the boreal midsummer in AD 774. It allowed for the produced ^14^C to be photosynthetically fixed during the late growing season of *P. sylvestris* in Finnish Lapland. This NH observation is in contrast with the one from the Southern Hemisphere, for which an event timing of spring/AD 775 has been proposed^7^, but is in agreement with estimations based on ^10^Be data of AD 774 occurrence^34^. Taking into account the uncertainties in the tropopause height profile and the assumed average atmospheric oxidation time of ^14^C of 1–2 months^35^, the solar event itself occurred few months earlier.

## Discussion

In summary, the ME has been observed in tree-rings of *P. sylvestris* from Finnish Lapland, so far closest to the GNP of AD 775. This has allowed us to demonstrate a latitudinal trend within the available data sets. By utilizing a peak-fitting approach, we find strong correlation between the latitude and determined ^14^C intensities. This is consistent with the assumed solar origin of the event. The timing is confirmed by sub-annual measurements of tree-ring cellulose showing anomalous increase of the ^14^C content in the LW of AD 774. This study illustrates the importance of trees growing near the boreal tree limit for storing information regarding both space weather and atmospheric circulation patterns.

## Methods

### ^14^C production due to an SPE

The production of ^14^C was calculated using the new-generation model^36^, which simulates, using a full Monte-Carlo technique, nucleonic cascade induced by cosmic rays in the Earth’s atmosphere and yields a 3D distribution of the ^14^C production. The spectrum of energetic particles for the event was considered hard^2,12^. The geomagnetic field was considered according to the archaeomagnetic model by Licht et al.^11^.

As an estimate of the tropospheric production, we applied a flat mean tropopause at the height of 150 hPa (cf. Usoskin et al.^2^) and found that about 30% of polar ^14^C is produced in the troposphere, for a hard-spectrum SPE. We note that globally about half of ^14^C is produced in the troposphere for such event. However, the flat-troposphere assumption may not exactly hold for the polar region, where the tropopause is typically lower, at the 200–300 hPa barometric pressure level, leading to a lower tropospheric production. When applying a local height profile of the polar tropopause^37,38^, we found that the fraction of tropospheric production of ^14^C in the polar region is about 15%. Accordingly, we used this range as an uncertainty and translated it into the uncertainty of the event’s date derivation.

The map of the study region (Fig. 1) was created using Matlab R2016b software (https://www.mathworks.com/). Specifically, the *R*_C_ values from Supplementary Fig. 1 and the respective ^14^C production values from Supplementary Table 2 were used to define the differently coloured regions. The GNP and the measurement locations are given in Supplementary Table 1. The data and the code to reproduce the figure can be obtained from the authors.

### Estimation of vertical cutoff rigidities

Although the ME took place well before the era of direct geomagnetic measurements, we are able to assess the geomagnetic cutoff rigidities at different locations for the period relying on precise archaeomagnetic reconstructions. These are based on measurements of the archaeologically dated clay samples preserving the local magnetic intensity during the time of their firing. Since the eighth century was rich for archaeological artefacts, the quality of geomagnetic field reconstructions is reasonable for that period.

Here we use the AF_M archaeomagnetic reconstruction model^11^, which is provided as an ensemble of 1000 individual reconstructions of the geomagnetic field with a pseudo annual resolution. The ensemble naturally covers all the sources of uncertainties of the reconstruction (measurement errors, sample size, systematic errors, model uncertainties).

For each of the individual ensemble member reconstructions we calculated, for a given location, the geomagnetic cutoff rigidity *R*_C_, using the eccentric dipole approximation of the field, based on the first eight Gaussian spherical coefficients (see full details in the Appendix A of Usoskin et al.^39^). The mean *R*_C_, and its standard deviation, was finally calculated from the obtained 1000 values of individual *R*_C_s over the ensemble, as shown in Supplementary Table 1.

We choose the AF_M model because it provides a full ensemble to assess the error bars and the Gaussian coefficients, and this result is consistent, within the shown uncertainties, with the geomagnetic dipole moment provided by other recent archeomagnetic reconstructions for the period of AD 775^36,40–44^.

In the same way, we calculated a map of the *R*_C_ values for the AD 775 epoch (Supplementary Fig. 1), using the mean *R*_C_ values.

### Tree-ring and radiocarbon analyses

A subfossil sample with well discernible rings over the study period was chosen for this analysis. The sample was tree-ring dated using statistical routines^45^ and visually by comparing the series of its ring widths against those of the master chronology^14^. The widths of the annual rings were on average 0.49 mm, with maximum and minimum widths of 0.73 and 0.37 mm, respectively. The sampling was done using a sterile surgical blade under the light microscope. No cross-contamination from one ring to the next was allowed by visual inspection. The separation of the EW and LW of the same calendar year was based on the intra-annual cellular characteristics discernible on the cross-sectional surface of the sample, generally following the established criteria^46^. In tree-ring laboratory, all the work to extract the isotope samples was done on cleaned sample surfaces to minimize any potential external contamination.

Tree-ring dated wood slivers were processed to α-cellulose using the batch-approach designed by Wieloch et al.^47^. The process consists of two alkaline extractions (5–7% NaOH), with a chlorination step (NaClO_2_) in between. The resulting α-cellulose was homogenized using an ultrasonic probe^48^ and freeze-dried. Pretreated samples were mixed with a stoichiometric excess of CuO and packed into quartz ampoules, which were evacuated and torch-sealed. The packed samples were combusted at 850 °C overnight. The released CO_2_ was collected and purified with liquid N_2_ and ethanol traps at −196 and −85 °C, respectively. The CO_2_ samples were converted to graphite targets^49^ for AMS radiocarbon measurements. AMS measurements were eventually performed at the University of Helsinki AMS facility^50^. The results are given as age-corrected Δ^14^C values^51^. For more detailed description of the analyses, see Helama et al.^52^.

### Peak analysis procedure and fittings

Stratospheric mean residence times of ^14^C are typically 1–2 years. Therefore, it can be estimated that after, say 3 years, 5–22% of ^14^C remains still in stratosphere. Thus, stratosphere supplies new ^14^C into troposphere, and therefore for photosynthesis, several years after an abrupt event. Peak-fitting approach was chosen since it allows for quantifying the peak shape by taking into account all the available information, namely the data and its uncertainties, during the time span of this ^14^C supply. In addition, our approach takes into account the possible laboratory biases caused by slightly different pre-treatment procedures. These biases are typically systematic and could cause some differences in the observed baseline levels. Our method helps to mitigate this effect, since it takes into account the respective baseline of the data. Furthermore, the full peak shape contains also information on possible use of formed year photosynthetic storages.

We fitted a Type-1 Gumbel distribution function (GDF) to each of the data. The GDF has the following form:

1 $$y = y_0 + A{\mathrm{e}}^{\left( { - {\mathrm{e}}^{\left( { - \frac{{x - x_{\mathrm{c}}}}{w}} \right)} - \frac{{x - x_{\mathrm{c}}}}{w} + 1} \right)},$$

where *y*_0_ is the baseline level (also used when adjusting the peaks to zero baseline), *A* is the amplitude of the peak, *x*_c_ is the time of the peak maximum and *w* is the width of the peak. GDF is normally used in extreme event statistics^53^ and its shape is characterized by a rapid rise and an exponential tail. Therefore, it is suitable for deducing intensities of rapidly increasing and slowly decreasing events occurring together with a relatively constant background, such as ^14^C intensity increase due to an SPE. Other distributions with similar shapes, such as Log-Normal and Exponentially Modified Gaussian that are often used in peak analysis^54^ were also considered, but GDF was found to be superior because of its overall convenience of use, simplicity of equation and property to not over-fit to statistical noise. Furthermore, GDF has been used before in peak analyses in a similar fashion to what is done here^55^. The GDF fit to each of the ME measurement can be seen in Supplementary Figs 3–10. Additionally, Supplementary Fig. 11 shows a GDF fit to a typical Δ^14^C profile due to instant injection of ^14^C into the atmosphere modelled by our adopted CCM.

To get the peak intensities, we analytically integrate the GDF in Eq. (1), which then becomes

2 $$y = {\int} {A{\mathrm{e}}^{\left( { - {\mathrm{e}}^{\left( { - \frac{{x - x_{\mathrm{c}}}}{w}} \right)} - \frac{{x - x_{\mathrm{c}}}}{w} + 1} \right)}} {\mathrm d}x = Aw{\mathrm{e}}^{\left( {1 - {\mathrm{e}}^{\left( {\frac{{x_{\mathrm{c}} - x}}{w}} \right)}} \right)} + C.$$

The interval used to calculate the integral for each of the data sets is bound to be from 770 to 779. The interval was chosen since it covers the event occurrence and the tropospheric near-event time distribution of ^14^C but not the long tail influenced merely by carbon cycle characteristics. Thus, the equation for the ^14^C intensity *I*_14C_ becomes

3 $$I_{14{\mathrm{C}}} = \mathop {\smallint }\limits\int_{770}^{779} A{\mathrm{e}}^{\left( { - {\mathrm{e}}^{\left( { - \frac{{x - x_{\mathrm{c}}}}{w}} \right)} - \frac{{x - x_{\mathrm{c}}}}{w} + 1} \right)}{\mathrm d}x = Aw{\mathrm{e}}^{\left( {1 - {\mathrm{e}}^{\left( {\frac{{x_{\mathrm{c}} - 779}}{w}} \right)}} \right)} - Aw{\mathrm{e}}^{\left( {1 - {\mathrm{e}}^{\left( {\frac{{x_{\mathrm{c}} - 770}}{w}} \right)}} \right)}.$$

In addition, an error estimate *σ* of the *I*_14C_ is calculated. This is done by propagating errors for the known values of *A*, *x*_c_, *w* and their standard errors in Eq. (3). The integrated ^14^C intensities can be seen in Supplementary Table 1.

To be confident on the capability of the peak-fitting method to capture the underlying ^14^C intensity, we tested our method by comparing simulated and peak-fitted peak sizes to the theoretically expected ones (Supplementary Fig. 2). The CCM provides a theoretically expected ^14^C intensity (red line in Supplementary Fig. 2) based on the underlying ^14^C production. The Monte Carlo simulation provides ^14^C peaks (10^5^ runs) for each underlying ^14^C intensity by assuming 3% statistical measurement errors. These were peak-fitted individually to obtain simulated ^14^C intensities (open squares in Supplementary Fig. 2). This sensitivity analysis demonstrated that the peak-fitting method captures the underlying relative ^14^C intensities extremely well. Hence, the peak-fitting method can be considered robust.

### ^14^C intensities in NH1 and non-NH1 zones

Atmospheric ^14^C analyses based on bomb peak have demonstrated distinct zones of varying ^14^C contents within the NH^18^. These are related to the observed circulation cells within the atmosphere. Visually, the ^14^C intensities of NH1 and non-NH1 zones for ME seem to form two latitudinally separate groups (Fig. 3). We tested whether this difference is statistically meaningful. Because the individual ^14^C intensities do not necessarily follow a normal distribution, we could not use Student’s *t*-test or any other test that assumes normality. Hence, we used a resampling method to perform an exact significance test. Specifically, this was done as follows. First, we calculated the test statistic (e.g., difference of means) using groups NH1 and non-NH1. Second, we combined the values of NH1 and non-NH1 into a single pool. Third, we performed a Monte Carlo simulation (10^5^ runs) where we randomly recombined these values into two groups with sizes of NH1 (5) and non-NH1 (4) groups. Fourth, we calculated the probability (*p*-value) of finding values more extreme than our test statistic. The above analysis was performed using both the “difference of means between group1 and group2” and the “sum of variances of group1 and group2” as test statistics. In both cases, the probability of finding value as extreme or more extreme than our test statistic is *p* = 0.008. This analysis shows it is unlikely that the observed ^14^C intensities of NH1 and non-NH1 groups originate from the same probability distribution, thus indicating latitudinal differences.

### Carbon cycle model

We adopted a 11-box CCM from Güttler et al.^7^. Their model has an advantage of having a resolution of 1 month instead of 1 year used in most studies regarding the ME. Hence, it is especially suitable in assessing the timing of the event.

The carbon reservoir masses (a unit corresponds to 10^12^ kg) and the annual fluxes (10^12^ kg yr^−1^) between them can be seen in Supplementary Fig. 12. A mean atmospheric ^14^C production rate of 1.88 ^14^C cm^−2^ s^−1^ atoms, which corresponds to an excess of 7.0 kg yr^−1^, was used to obtain the atmospheric ^14^C concentrations at around AD 775. In our model runs, we assume an event production rate of 83 ^14^C cm^−2^ s^−1^ over 1 month totalling to 25.8 kg of ^14^C. Seventy percent of the total ^14^C production is assumed to have taken place in the stratosphere and 30% in the troposphere, which is also in line with Usoskin et al.^2^.

The model assumes that the ^14^C is in the form of ^14^CO_2_, which is not true immediately after the ^14^C production. Although ^14^CO is formed very rapidly, the mean oxidation time *τ* for ^14^CO is approximately 1–2 months^35^. Since oxidation is a statistical process, some ^14^CO molecules get oxidized almost immediately, whereas it takes a long time for all the molecules to be oxidized. The rate of oxidation can be calculated using the exponential decay equation $N\left( t \right) = 1 - {\mathrm{e}}^{ - \frac{t}{\tau }}$, where *t* is the elapsed time and *τ* is the mean oxidation time. Assuming *t* = *τ* = 2 months, we find that $1 - \frac{1}{{\mathrm{e}}}$ (~2/3) of the initially produced ^14^C has oxidized to ^14^CO_2_. Hence, we define our use of word timing to mean the moment, when $1 - \frac{1}{\mathrm e}$ of the originally produced ^14^C has oxidized to ^14^CO_2_. In addition to being compatible with the model output, this definition is compatible with tree-ring measurements, since most of the original ^14^C is in a form that can be photosynthetically sampled by trees. However, it is noted that the non-zero mean oxidation time adds up to 2 months systematic uncertainty as to when the initial ^14^C production occurred.

### Growing seasons and TTS

To estimate whether the climate around AD 775 was different from the modern era, we made a comparison between (a) the year AD 1959–2015 average *T*_July_ (Finnish Meteorological Institute data, http://en.ilmatieteenlaitos.fi/open-data-manual) for Inari (68.9N, 27.0E) and (b) the tree-ring width-based reconstructed temperatures of Finnish Lapland during AD 750–800^56^. The averages were *T*_July 1959–2015_ = 13.7 ± 1.8 °C and *T*_July, rec 750–800_ = 13.1 ± 0.9 °C. These values are identical within the uncertainties. Thus, we assumed similarity of the climate and thus the growing seasons for the era of the ME and of modern times.

To mimic the actual tree growth mediated by photosynthetic assimilation during the growing season within our model, we took into account the average (AD 1959–2015 from Inari) TTS and weighted the monthly ^14^C concentration (given by the model) by monthly fractions of TTS to obtain the peak shape in tree rings. These values can be seen in Supplementary Fig. 13.

### Timing of the event by carbon cycle modelling

To estimate the timeframe for the event occurrence, we analyzed the data of the northernmost location LAP in more detail. The adopted CCM reproduces reasonably well the observed individual ^14^C peak shapes (Supplementary Figs. 3–10). Moreover, we can reproduce the observed changes in the ^14^C intensities with the model. Therefore, we adopted the modelled peak shape also for comparison to estimate the timeframe of the event. We weighted the modelled tropospheric ^14^C content within the assumed growing season by monthly TTS to get an average ^14^C content of tree rings for each year (AD 770–779). These modelled data, assuming different occurrence times of the event with a monthly resolution, were then compared with the measured data to find the lowest RSS, which is defined as follows:

4 $${\mathrm{RSS}} = \mathop {\sum }\limits_{i = 1}^n \left( {y_{i} - f(x_{i})} \right)^2,$$

where *y*_*i*_ is the measured Δ^14^C and *f*(*x*_*i*_) is the modelled Δ^14^C for each year through 770–779. These RSS values can be seen in Supplementary Fig. 14.

### Data Availability

The data that support the findings of this study are available from the corresponding author upon reasonable request.

## Electronic supplementary material

Supplementary Information
