# Author Correction: Solar superstorm of AD 774 recorded subannually by Arctic tree rings

**DOI:** 10.1038/s41467-019-09214-w

**Published:** 2019-03-15

**Authors:** J. Uusitalo, L. Arppe, T. Hackman, S. Helama, G. Kovaltsov, K. Mielikäinen, H. Mäkinen, P. Nöjd, V. Palonen, I. Usoskin, M. Oinonen

**Affiliations:** 1 Finnish Museum of Natural History, University of Helsinki, P.O. Box 64, 00014 Helsinki, Finland; 2 Department of Physics, University of Helsinki, P.O. Box 64, 00014 Helsinki, Finland; 3 Natural Resources Institute Finland, Eteläranta 55, 96300 Rovaniemi, Finland; 4 Ioffe Physical-Technical Institute, Politekhnicheskaya 26, 194021 St. Petersburg, Russia; 5 Natural Resources Institute Finland, Tietotie 2, 02150 Espoo, Finland; 6 Space Climate Research Unit and Sodankylä Geophysical Observatory, University of Oulu, Pentti Kaiteran katu 1, 90014 Oulu, Finland

Correction to: *Nature Communications* (2019); 10.1038/s41467-018-05883-1; published online 28 August 2018

The authors became aware of a mistake in the data displayed in Fig. 1 and Supplementary Table 2 of the original version of the Article. Specifically, the ^14^C production values were printed out in the code before the conversion between the omnidirectional fluence and the flux. As a consequence, the values of the ^14^C production in Fig. 1 and Supplementary Table 2 were too high by a factor of 4 × *π* = 12.566.

As a result of this, the following changes have been made to the originally published version of this Article:

In the original version of Fig. 1, the labels on the colour scale bar were incorrect. The previous incorrect version read, from top to bottom ‘1.40E + 03, 3.25E + 03, 8.00E + 03, 2.70E + 04, 6.00E + 04, 1.50E + 05, 1.75E + 06, 8.50E + 06, 5.25E + 07, 1.80E + 08, 2.29E + 08’. The corrected figure replaces these with, from top to bottom ‘1.11E + 02, 2.59E + 02, 6.37E + 02, 2.15E + 03, 4.77E + 03, 1.19E + 04, 1.39E + 05, 6.76E + 05, 4.18E + 06, 1.43E + 07, 1.82E + 07’.

In the original version of Supplementary Table 2, the values in the second column were incorrect. The correct version of Supplementary Table 2 is:

**Table:** 

*R*_*C*_ (GV)	^14^C production (atoms/cm^2^)
0	1.82E + 07
0.2	1.82E + 07
0.7	1.43E + 07
1.4	4.18E + 06
2.4	6.76E + 05
3.5	1.39E + 05
6.1	1.19E + 04
7.5	4.77E + 03
8.9	2.15E + 03
11.7	6.37E + 02
14.1	2.59E + 02
17.0	1.11E + 02

which replaces the previous incorrect version:

**Table:** 

*R*_*C*_ (GV)	^14^C production (atoms/cm^2^)
0	2.29E + 08
0.2	2.29E + 08
0.7	1.80E + 08
1.4	5.25E + 07
2.4	8.50E + 06
3.5	1.75E + 06
6.1	1.50E + 05
7.5	6.00E + 04
8.9	2.70E + 04
11.7	8.00E + 03
14.1	3.25E + 03
17.0	1.40E + 03

This has been corrected in both the PDF and HTML versions of the Article. This error does not affect the main result, original discussion or conclusions of the Article.

