# Supplementary material for: Solar superstorm of AD 774 recorded subannually by Arctic tree rings
Source: Nat Commun. 2018 Aug 28;9:3495. doi: 10.1038/s41467-018-05883-1 (PMC6113262; doi:10.1038/s41467-018-05883-1)
Supplement: Supplementary file 1 — Supplementary Information [file 41467_2018_5883_MOESM1_ESM.pdf]

Supplementary Information

Solar superstorm of AD 774 recorded subannually by Arctic tree rings

Uusitalo et al.

Supplementary Figures 1-14

Supplementary Tables 1-3

Supplementary Notes 1-2

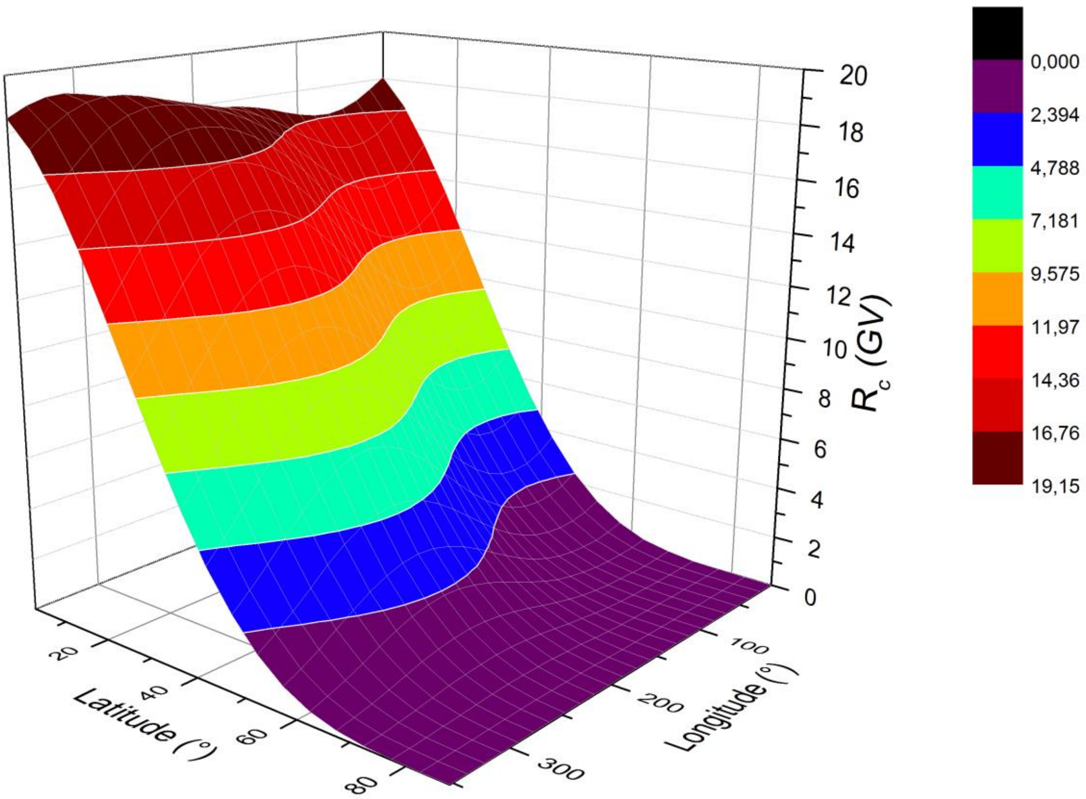

Supplementary Figure 1. Vertical geomagnetic cutoff rigidities at AD 775.

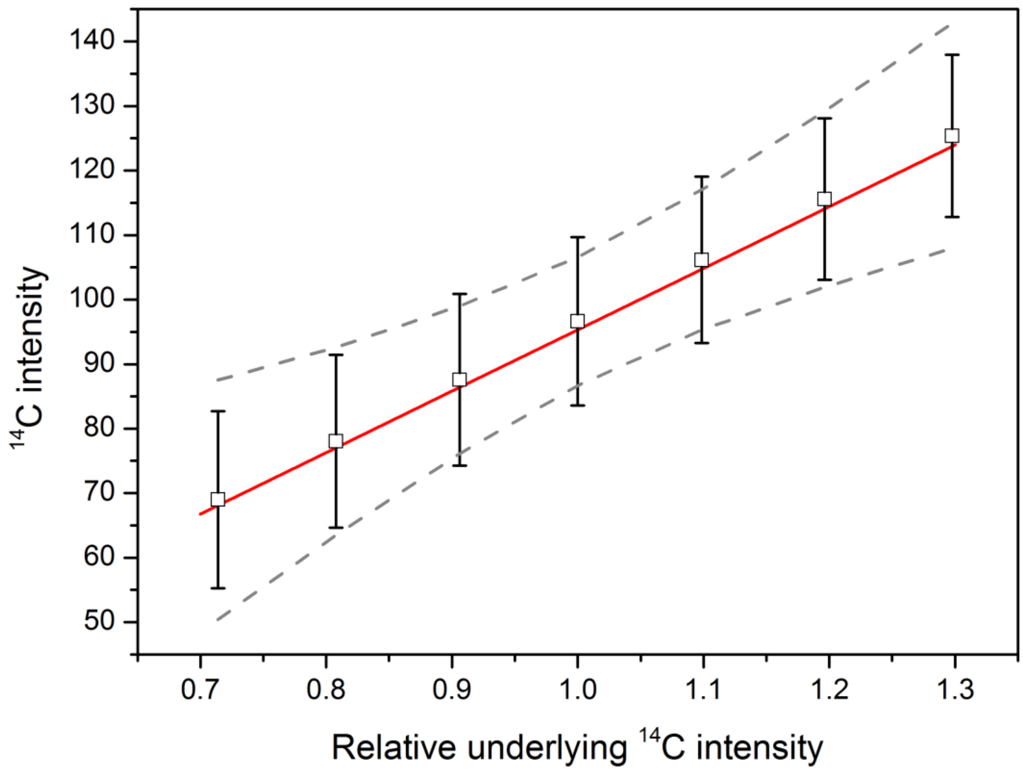

Supplementary Figure 2. Test for the peak fitting method to statistically find a trend in ^{14}C intensities. The red line shows the theoretically expected ^{14}C intensity in relation to the underlying ^{14}C intensity. The open squares show the average of the Monte Carlo simulated ^{14}C intensities and their standard deviation. The dashed gray lines show the 95% confidence limits for the linear fits generated using the simulated data.

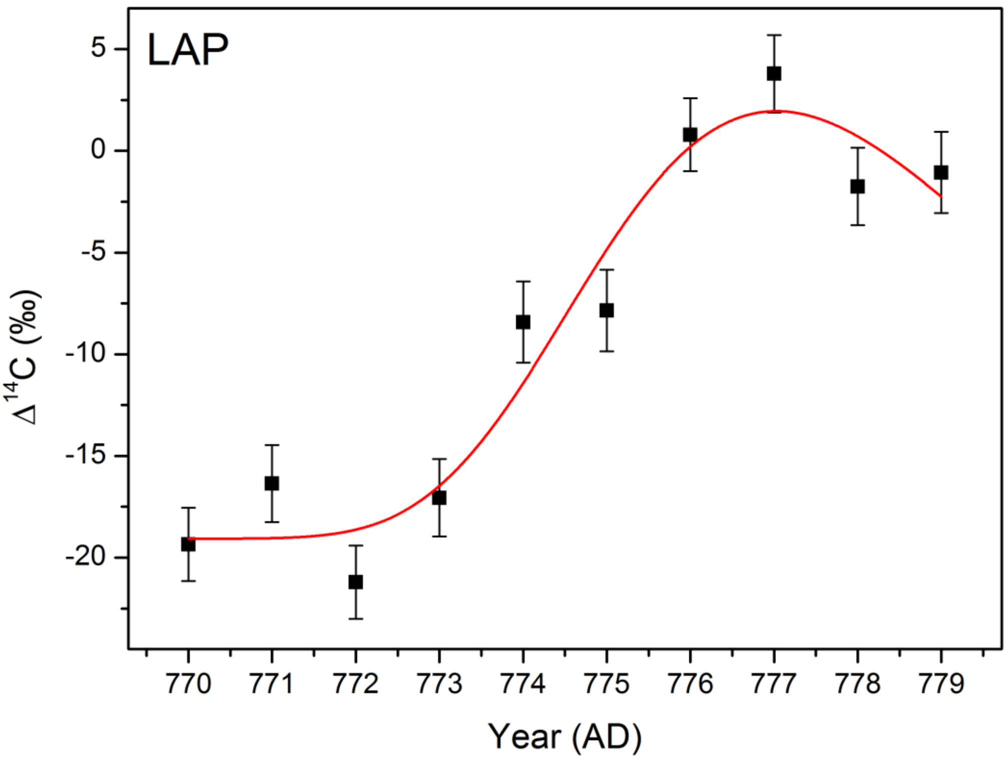

Supplementary Figure 3. GDF fit to the LAP ^{14}C data. Adjusted r^2 = 0.92. The error bars represent one standard error.

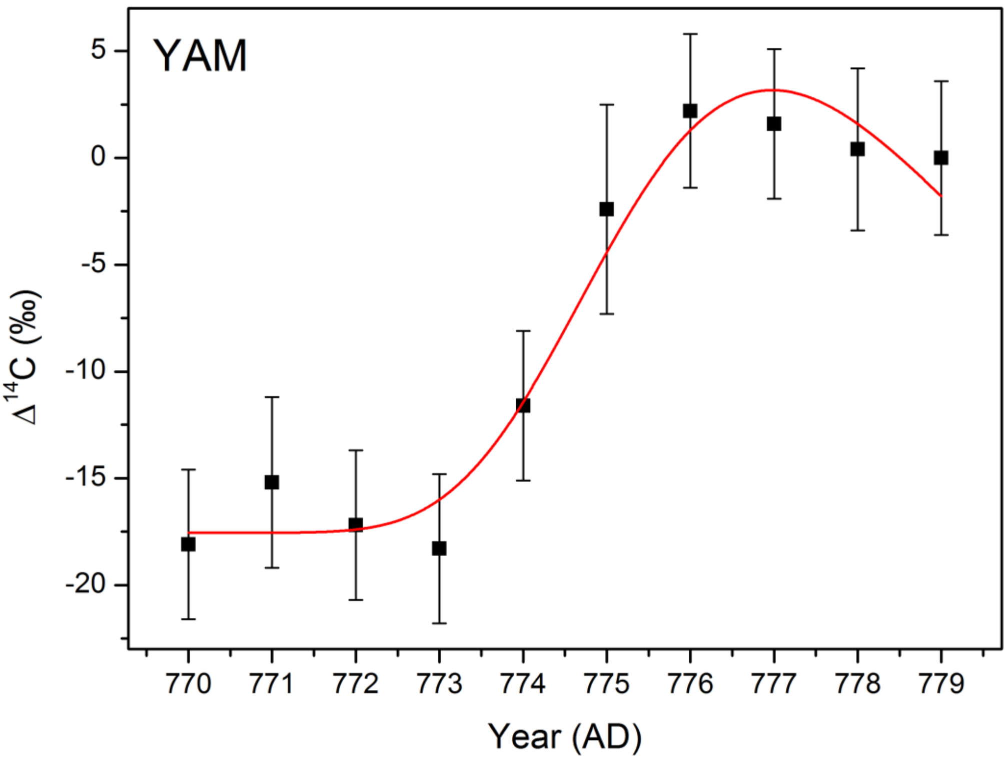

Supplementary Figure 4. GDF fit to the YAM ^{14}C data. Adjusted r^2 = 0.96. The error bars represent one standard error.

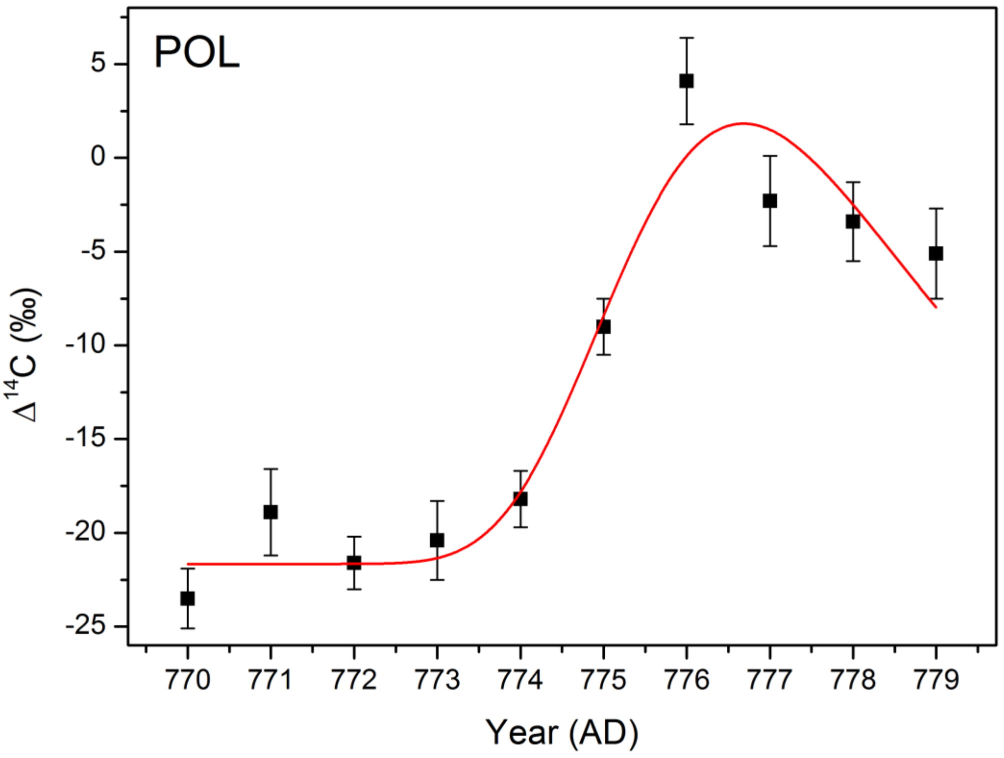

Supplementary Figure 5. GDF fit to the POL ^{14}C data. Adjusted r^2 = 0.93. The error bars represent one standard error.

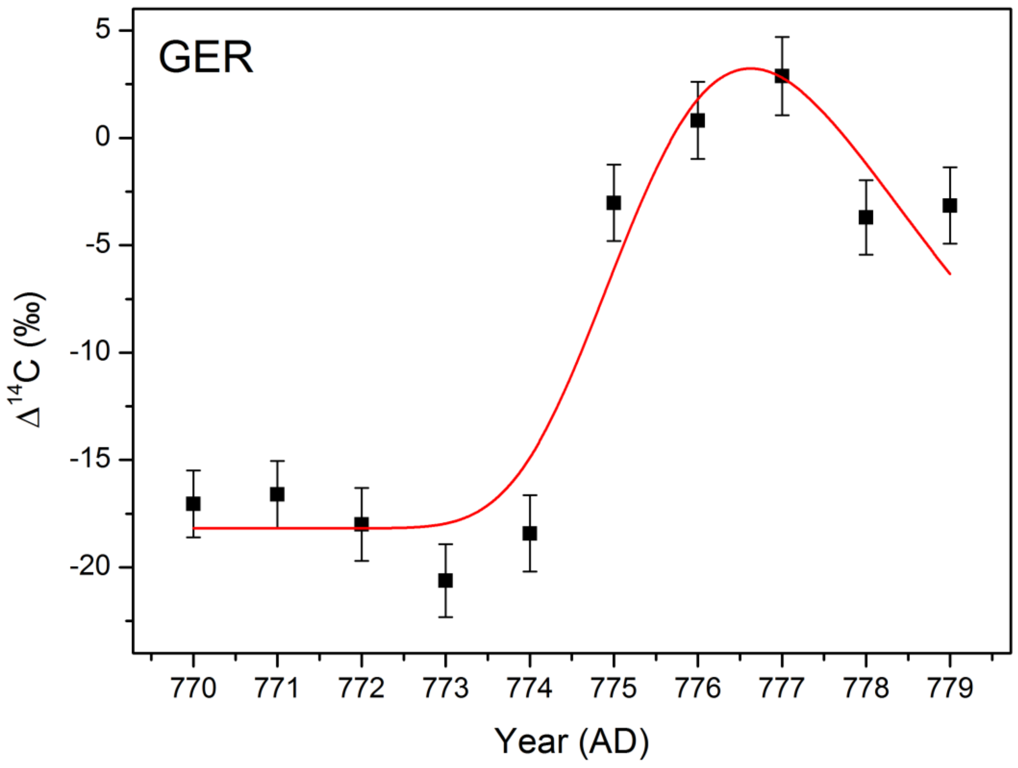

Supplementary Figure 6. GDF fit to the GER ^{14}C data. Adjusted r^2 = 0.90. The error bars represent one standard error.

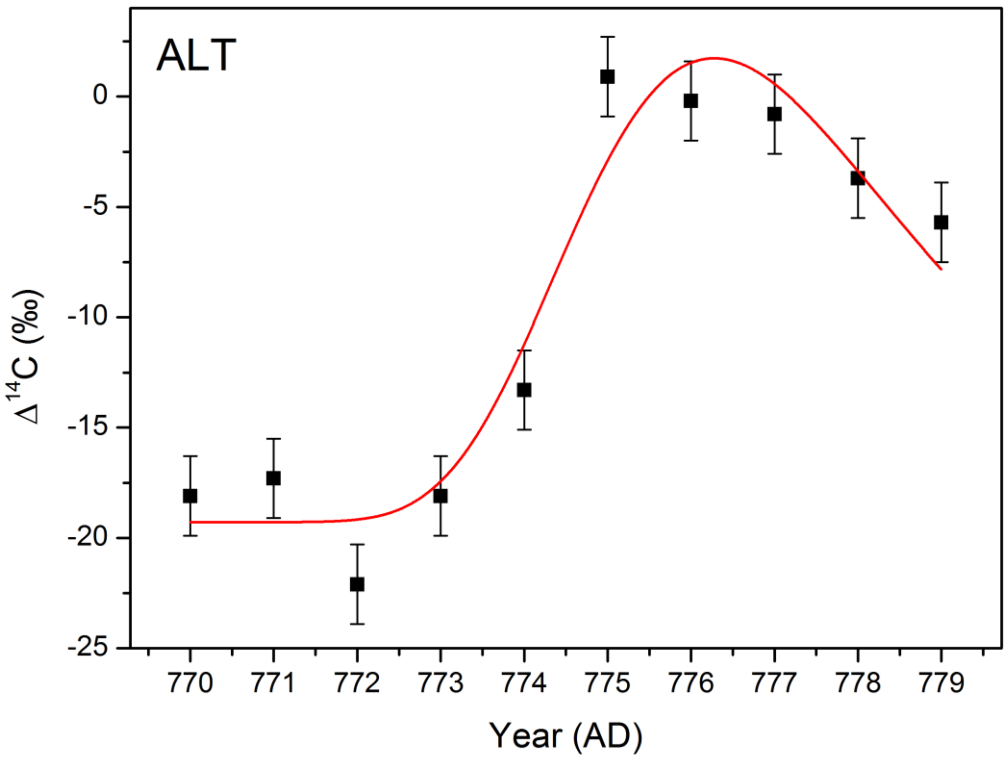

Supplementary Figure 7. GDF fit to the ALT ^{14}C data. Adjusted r^2 = 0.91. The error bars represent one standard error.

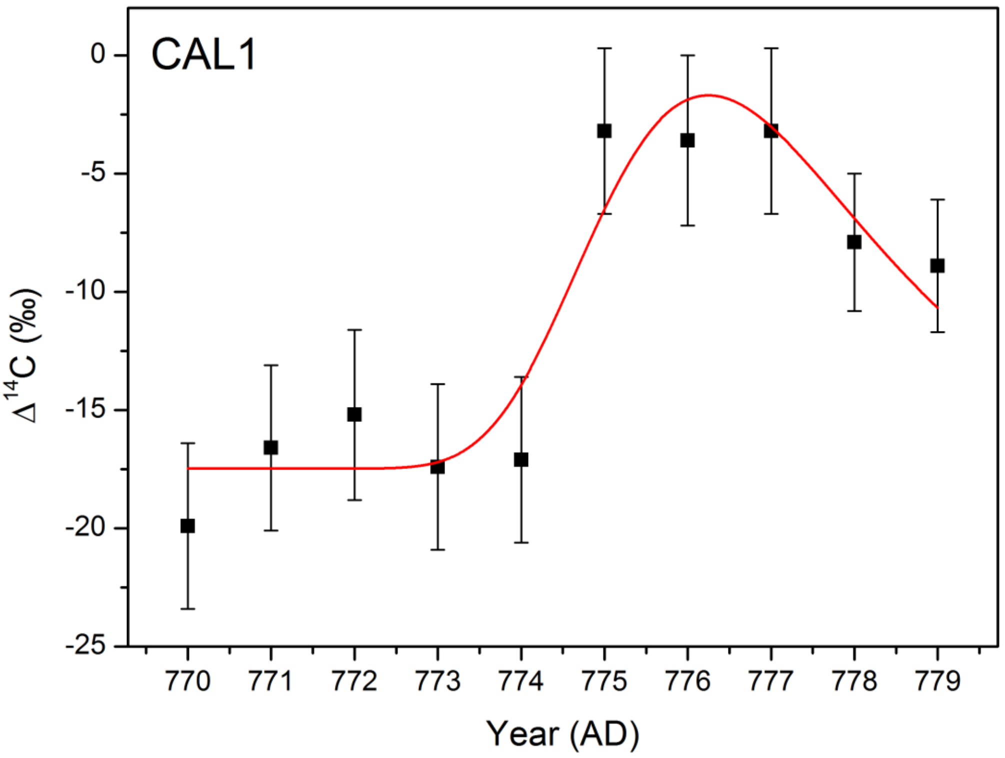

Supplementary Figure 8. GDF fit to the CAL1 ^{14}C data. Adjusted r^2 = 0.84. The error bars represent one standard error.

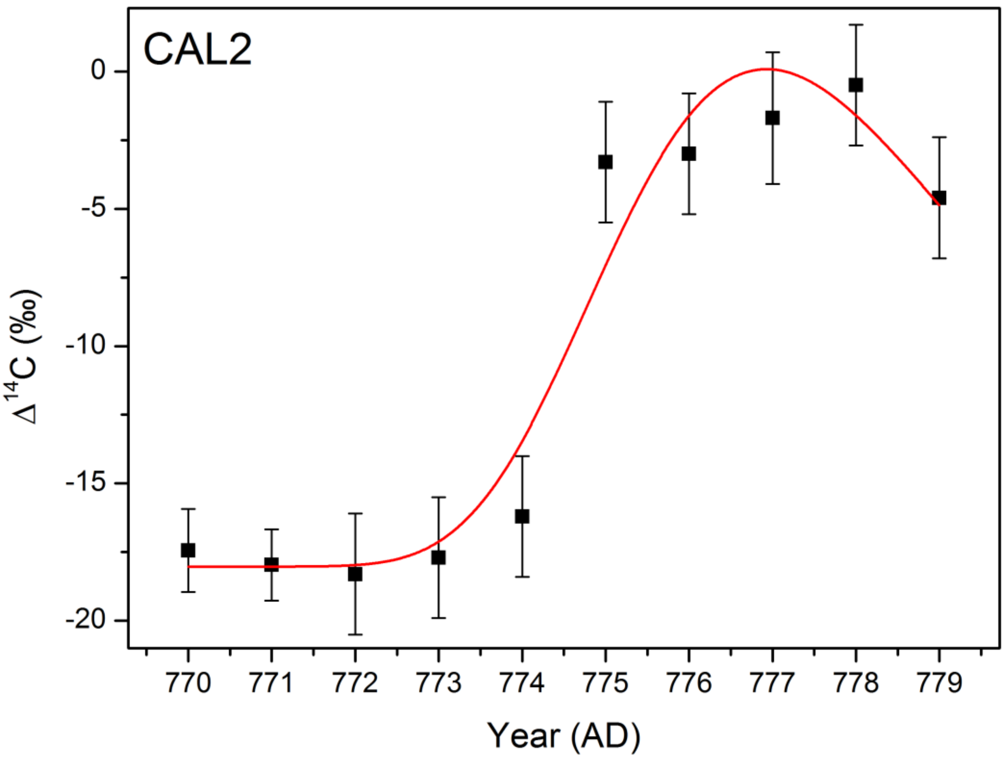

Supplementary Figure 9. GDF fit to the CAL2 ^{14}C data. Adjusted r^2 = 0.94. The error bars represent one standard error.

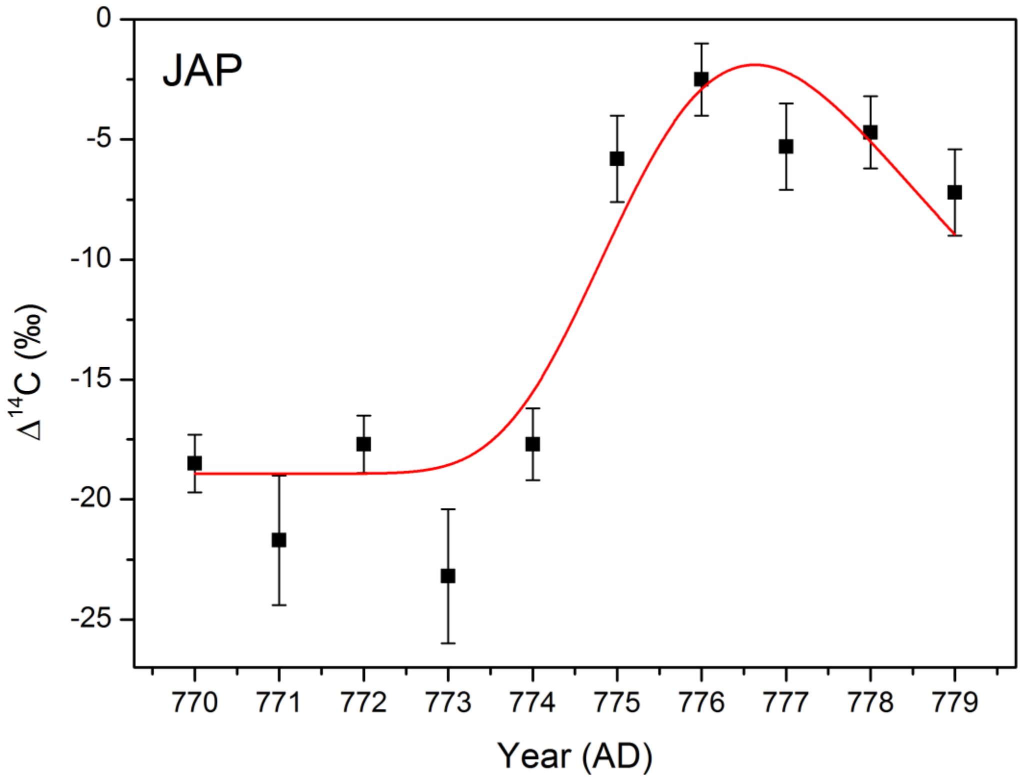

Supplementary Figure 10. GDF fit to the JAP ^{14}C data. Adjusted r^2 = 0.89. The error bars represent one standard error.

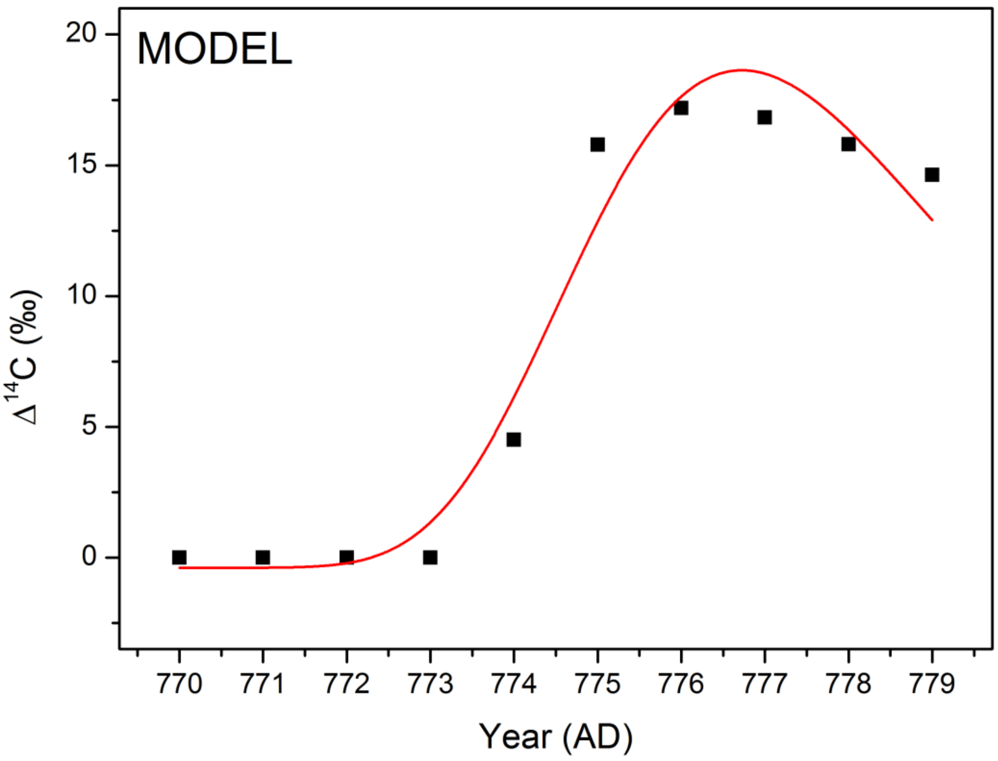

Supplementary Figure 11. Example of a GDF fit to a carbon cycle model output with an assumed event occurrence of 8/774 and a growing season from May to September. Adjusted r^2 = 0.95.

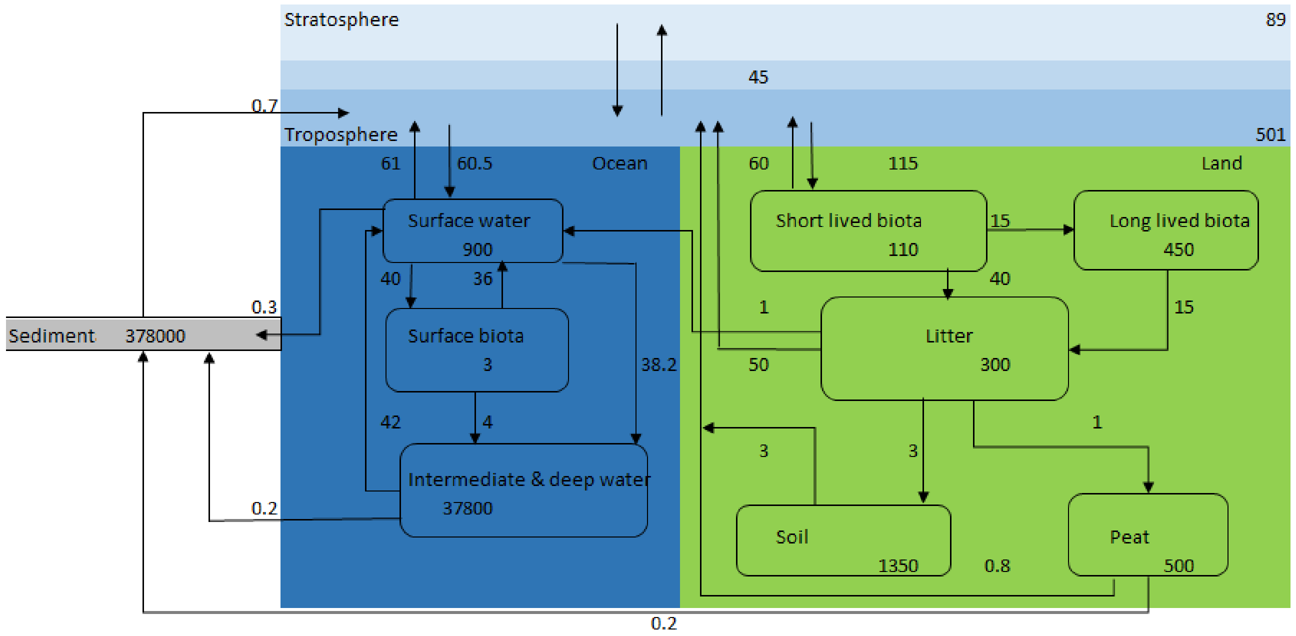

Supplementary Figure 12. 11-box carbon-cycle model. Illustration based on Güttler et al.[1]. The units of carbon reservoir masses and annual fluxes correspond to 10^{12} kg and 10^{12} kg yr^{-1}, respectively.

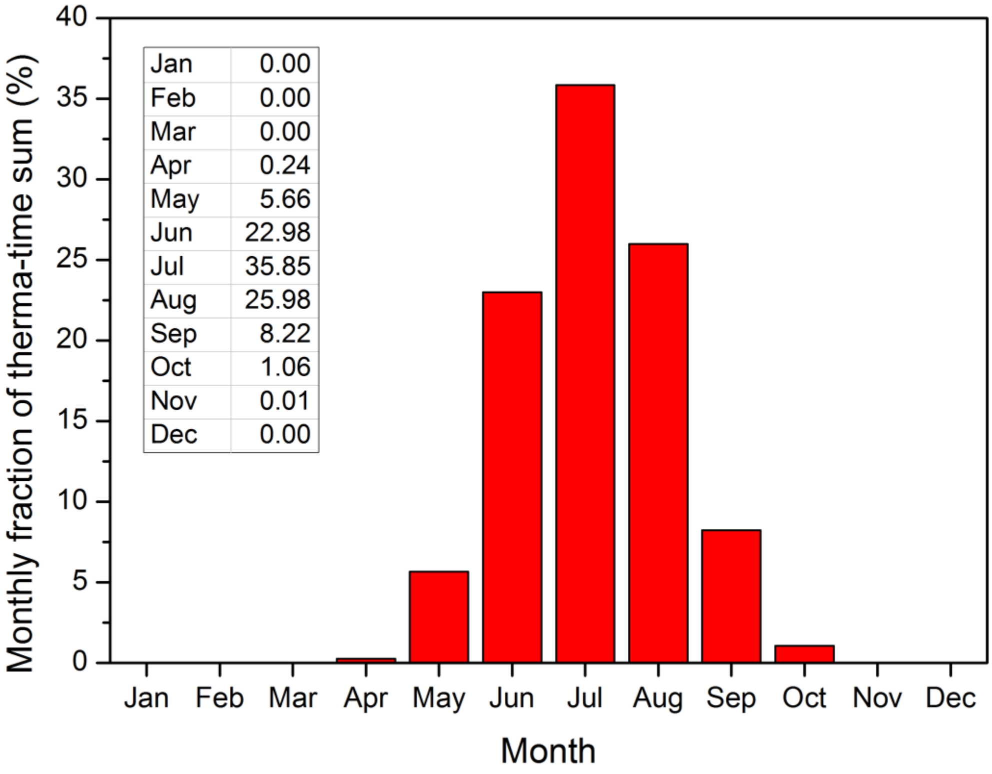

Supplementary Figure 13. The monthly fraction of the thermal time-sum for LAP.

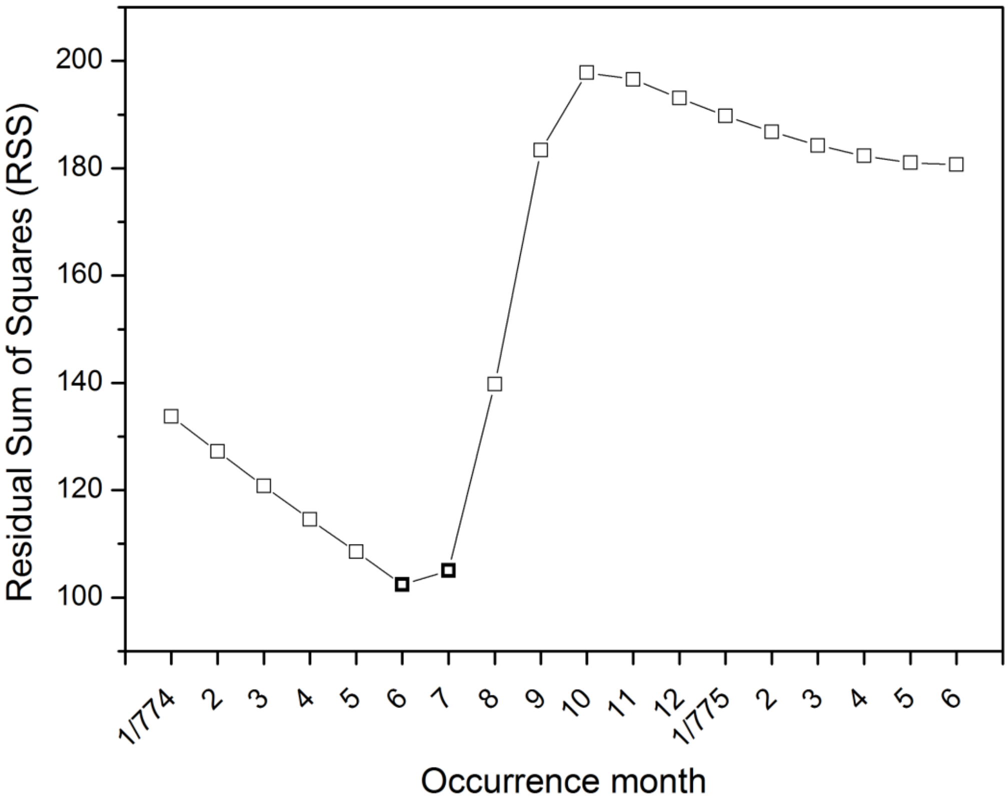

Supplementary Figure 14. Residual sum of squares (RSS) for different event occurrence months. Two bolded squares indicate the lowest RSS values observed (June-July/774).

Supplementary Table 1. The obtained ^{14}C intensities (I_{14C}) and modelled vertical cutoff rigidities (R_{C_AD775}) at the various locations together with the Pearson correlation coefficient ($r(R_{C\_AD775}, I_{14C})$).

	**Geodetic coordinates**	**R_C (AD775)**	**1σ error**	**I_{14C}**	**1σ error**
**GNP**	~82N, 25E	~0			
**LAP**	68.5N, 28.1E	0.07	0.02	93.7	9.2
**YAM**	67.5N, 70.7E	0.09	0.03	87.9	16.7
**POL**	50.1N, 20.1E	1.3	0.2	88.8	6.7
**GER**	50.0N, 9.0E	1.9	0.3	79.9	6.2
**ALT**	47.5N, 87.5E	2.3	0.3	89.0	8.0
**JAP**	30.4N, 130.5E	9.5	0.6	66.0	6.0
**CAL1**	38.3N, 118.7W	10.7	0.5	59.1	13.3
**CAL2**	36.5N, 118.8W	10.7	0.5	74.3	7.6
**NZL**	36.0S, 173.8E	-	-	70.5	6.5
**$r(R_{C\_AD775}, I_{14C})$**		0.87			
**p val**		0.002			

Supplementary Table 2. Modelled ^{14}C production due to a SPE of Feb-23, 1956 but for the geomagnetic conditions corresponding to AD 775.

**R_C (GV)**	**^{14}C production (atoms/cm^2)**
0	1.82E+07
0.2	1.82E+07
0.7	1.43E+07
1.4	4.18E+06
2.4	6.76E+05
3.5	1.39E+05
6.1	1.19E+04
7.5	4.77E+03
8.9	2.15E+03
11.7	6.37E+02
14.1	2.59E+02
17.0	1.11E+02

Supplementary Table 3. Annual, earlywood (EW), latewood (LW) radiocarbon measurements and tree-ring widths (RW) for northern Lapland subfossil Scots pine performed for this work.

Year	$\Delta^{14}C$(‰)	1σ	$\Delta^{14}C_{EW}$(‰)	1σ	$\Delta^{14}C_{LW}$(‰)	1σ	RW_{EW}(mm)	RW_{LW}(mm)
770	-19.4	1.8	-20.7	1.5	-24.3	1.6	0.32	0.09
771	-16.4	1.9	-20.4	1.6	-19.5	1.6	0.31	0.10
772	-21.2	1.8	-23.2	1.5	-15.3	1.5	0.41	0.08
773	-17.1	1.9	-18.9	1.5	-16.0	1.5	0.48	0.12
774	-8.4	2.0	-18.8	1.5	-9.1	1.5	0.31	0.10
775	-7.8	2.0	-11.2	1.5	-0.6	1.6	0.38	0.08
776	0.8	1.8	1.6	1.5	-1.9	1.6	0.38	0.09
777	3.8	1.9	-0.4	1.5	2.6	1.6	0.41	0.17
778	-1.8	1.9	-3.0	1.5	-8.8	1.5	0.70	0.26
779	-1.1	2.0	-5.9	1.5	-1.6	1.5	0.72	0.17

Supplementary Note 1. Effect of possible seasonal variation

We estimated the role of possible seasonal variation of tropospheric ^{14}C concentration into the deduced I_{14C} as follows. First, we used tropospheric ^{14}C measurement data of the bomb peak with 1-month resolution from Vermunt, Germany[2]. Second, we sampled this data year-by-year from 1959 to 1967 according to the estimated growing season of LAP (June-August) and JAP (May-October) locations to obtain yearly effect of the variation. Third, we created a modified Miyake event peak that takes into account these variations and compared it with the original LAP measurement. Our analysis shows that the effect of seasonality to the sampled ^{14}C values is strongest (i.e. ~5% difference) during the peak maximum and gets gradually weaker afterwards. The total estimated effect of seasonality to the JAP ^{14}C intensity in comparison to the LAP ^{14}C intensity is estimated to be ~3%. If a more extreme growing season difference of June-July vs. March-October is assumed, the effect is ~4%. Both of these estimates are well within the I_{14C} uncertainties. In comparison, the actual observed I_{14C} difference between LAP and JAP measurements is approximately 30%. Thus, we conclude that the effect of possible seasonal variation is insignificant.

Supplementary Note 2. Effect of uncertainties in tropopause height profile to the modelled timing

Considering that there is some uncertainty in the tropopause height profile, we performed sensitivity analyses by varying the stratosphere-troposphere ^{14}C production fractions from 85% to 55% and 15% to 45%, respectively. These analyses showed that assuming a stratosphere-troposphere production fractions of 85% and 15%, respectively, would prepone the most likely timing to April/AD 774, whereas fractions of 55% and 45%, would postpone it to July/AD 774.

Supplementary References

1. Güttler, D. *et al.* Rapid increase in cosmogenic 14C in AD 775 measured in New Zealand kauri trees indicates short-lived increase in 14C production spanning both hemispheres. *Earth Planet. Sci. Lett.* **411,** 290–297 (2015).

2. Levin, I. *et al.* Observations and modelling of the global distribution and long-term trend of atmospheric $^{14}CO_2$. *Tellus B Chem. Phys. Meteorol.* **62,** 26–46 (2010).
